# Rlim/Rnf12, Rex1, and X Chromosome Inactivation

**DOI:** 10.3389/fcell.2019.00258

**Published:** 2019-10-31

**Authors:** Feng Wang, Ingolf Bach

**Affiliations:** Department of Molecular, Cell and Cancer Biology, University of Massachusetts Medical School, Worcester, MA, United States

**Keywords:** mouse genetics, XCI, RLIM, Rnf12, Rex1, *Xist* regulation

## Abstract

RLIM/Rnf12 is an E3 ubiquitin ligase that has originally been identified as a transcriptional cofactor associated with LIM domain transcription factors. Indeed, this protein modulates transcriptional activities and multiprotein complexes recruited by several classes of transcription factors thereby enhancing or repressing transcription. Around 10 years ago, RLIM/Rnf12 has been identified as a major regulator for the process of X chromosome inactivation (XCI), the transcriptional silencing of one of the two X chromosomes in female mice and ESCs. However, the precise roles of RLIM during XCI have been controversial. Here, we discuss the cellular and developmental functions of RLIM as an E3 ubiquitin ligase and its roles during XCI in conjunction with its target protein Rex1.

## Introduction

X dosage compensation in female mice occurs early during embryogenesis in two waves. An early, imprinted form of XCI (iXCI), which silences exclusively the paternally inherited X (Xp), directly follows zygotic genome activation (ZGA) at the end of the 2-cell stage. While this pattern of XCI is maintained in extraembryonic tissues including trophoblast and primitive endoderm, epiblast cells which give rise to the embryo proper reactivate the Xp (XCR) and undergo a random form of XCI (rXCI) around implantation (Payer, 2016). The long non-coding (lnc) RNA *Xist* plays crucial roles during both forms of XCI and paints the X from which it is expressed (Brown et al., 1992; Penny et al., 1996). Initiation of *Xist* transcription is considered the onset of XCI and an early phase of continued *Xist* expression is required for the maintenance of the XCI state (Wutz and Jaenisch, 2000). Prior to upregulation of *Xist* during rXCI, which is mostly investigated in female ESC models, both X chromosomes transiently move into spatial proximity, a process known as X pairing (Bacher et al., 2006; Xu et al., 2006; Augui et al., 2007). *Xist* transcription is inhibited in *trans* by pluripotency transcription factors including Rex1 (Navarro et al., 2010) and in *cis* by the lnc RNA *Tsix*, which is transcribed antisense to *Xist* (Lee et al., 1999; Lee, 2000).

The *Rlim* gene (also known as Rnf12) encodes a RING finger ubiquitin ligase (E3) (Ostendorff et al., 2002). During mouse development *Rlim* mRNA is widely expressed, while RLIM protein expression is more restricted in cell types and tissues (Ostendorff et al., 2006). In cells, RLIM protein shuttles between the nucleus and cytoplasm in a phosphorylation-dependent manner (Jiao et al., 2013) but in most cell types, RLIM protein is detected in the nucleus, where many of its substrate proteins reside that include transcription factors and transcriptional co-regulators. Indeed, RLIM is involved in regulating the dynamics of DNA-bound multiprotein complexes in promoters/enhancers

(Ostendorff et al., 2002; Güngör et al., 2007; Johnsen et al., 2009). RLIM can self-ubiquitinate and mutations of the RING finger results in gain-of function activities and stabilization of the mutated protein (Ostendorff et al., 2002).

Important *in vivo* functions of *Rlim* have been discovered in female mice. In mammary glands of pregnant and lactating females, RLIM serves as a survival factor specifically for milk-producing alveolar cells (Jiao et al., 2012, 2013). Moreover, Rlim/Rnf12 has been identified as a major activator of XCI in female ESCs (Jonkers et al., 2009) and required for iXCI in female mice (Shin et al., 2010). In an ESC model RLIM interacts with Rex1/Zfp42, a transcriptional repressor of *Xist*, leading to proteasomal degradation and initiation of XCI (Gontan et al., 2012). However, *Rlim* was found dispensable for rXCI in epiblast tissues and in other ESC model systems (Shin et al., 2010, 2014), and thus over the last years there was much confusion on the roles and importance of *Rlim* during rXCI. Recent work has identified Rex1 as the critical *Rlim* target during iXCI and directly compared XCI in various ESC model systems (Wang et al., 2017; Gontan et al., 2018). Results illuminate major roles of *Rlim* in conjunction with Rex1 during XCI in nuclei of cells, thereby partially clarifying the existing controversy.

## RLIM as a RING Finger E3 Ubiquitin Ligase

**FIGURE 1:**
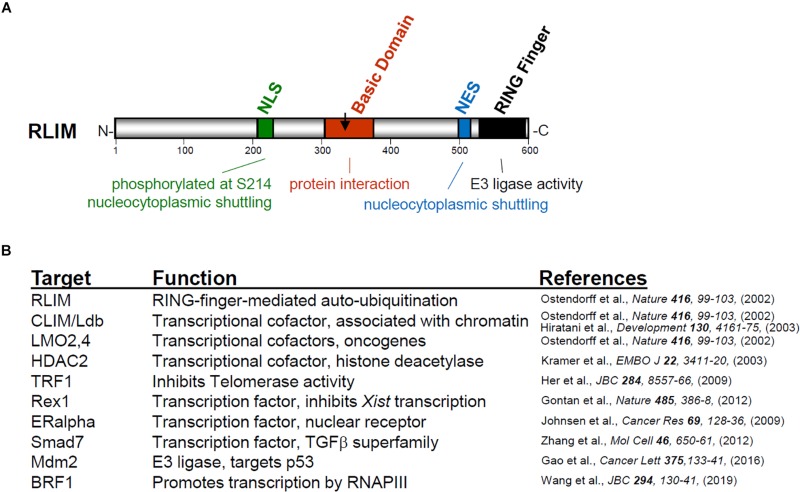
**(A)** Structure of RLIM protein indicating conserved domains, their functions and amino acid position. The arrow indicates the C-terminus of RLIM333. **(B)** Reported Rlim target proteins for ubiquitination. Note that most proteins are nuclear.

RLIM/Rnf12 was first identified as an antigen recognized by autologous antibodies of renal cancer patients (Scanlan et al., 1999) and as a cofactor negatively affecting the transcriptional and developmental activity of LIM homeodomain transcription factors (Bach et al., 1999). The gene *Rlim* maps to the X chromosome and is conserved from humans to chick (Ostendorff et al., 2000). RLIM protein in mice encompasses 600 amino acids (Figure 1A) and contains several conserved domains, including nuclear localization and export sequences (NLS and NES, respectively), a centrally located basic domain (BD) and a C-terminal RING-H2 zinc finger domain. Indeed, both the NLS and the NES are functional and rapid nucleocytoplasmic shuttling of the RLIM protein in a phosphorylation-dependent manner has been demonstrated (Jiao et al., 2013), even though in most cell types RLIM protein is detected predominantly in the nuclear compartment. The C-terminal RING H2 zinc finger motif identifies RLIM as an E3 ubiquitin protein ligase. RING finger E3 ligases are part of the ubiquitin proteasome system (UPS) involving an E1 activating enzyme, E2 ubiquitin-conjugating enzymes and E3 ubiquitin ligases which, by recognizing the substrate proteins, confer the specificity of the ubiquitination reaction (Metzger et al., 2014). Ubiquitinated proteins are often targeted for degradation by the 26S proteasome but this depends on mono-, multi- or poly-ubiquitination chains and the chain linkage type (Hicke, 2001; Pickart, 2001; Ikeda and Dikic, 2008; Metzger et al., 2014). Unlike the cullin subclass of RING finger E3s (Petroski and Deshaies, 2005), RLIM belongs to a subclass of RING finger E3s in which substrate recognition and ubiquitination ligase functions are mediated by the same protein (Metzger et al., 2012), and many substrate proteins of RLIM are recognized and bound by the Basic Domain (Figure 1A). As RLIM locates mostly to the nucleus, it is not surprising that substrates comprise mainly nuclear proteins, including LIM-only (LMO) and CLIM/Ldb cofactors (Ostendorff et al., 2002; Hiratani et al., 2003), the histone de-acetylase HDAC2 (Kramer et al., 2003), the telomeric protein TRF1 (Her and Chung, 2009), the oncoprotein Stathmin (Chen et al., 2014), estrogen receptor alpha (ERalpha) (Johnsen et al., 2009), the TFIIIB subunit BRF1 (Wang et al., 2019) and SMAD7 involved in TGFbeta signaling (Zhang et al., 2012). Due to its E3 ligase activity, RLIM regulates cellular levels of transcriptional co-regulators and is involved in the dynamics of multiprotein complexes on DNA in promoters/enhancers (Ostendorff et al., 2002; Güngör et al., 2007; Johnsen et al., 2009). Importantly, RLIM also targets Rex1 for proteasomal degradation (Gontan et al., 2012), a pluripotency factor expressed in early embryos (Climent et al., 2013) that acts as a transcriptional repressor of *Xist*. RLIM is able to self-ubiquitinate (Ostendorff et al., 2002), a hallmark of RING finger E3s, which likely contributes to its reported short half-life (Becker et al., 2003). Moreover, RLIM targets another RING E3 ligase Mdm2 for degradation (Gao et al., 2016) and is the target of the RING E3 ligase SIAH1/2 (Kramer et al., 2008). Indeed, as part of regulatory networks, both self-ubiquitination of RING finger E3s and ubiquitination of RING E3s by heterologous E3s frequently occurs *in vivo* (Weissman et al., 2011). These activities likely contribute to the observed differences in the widespread developmental *Rlim* mRNA expression, and the more restricted RLIM protein expression (Ostendorff et al., 2006).

The activity of E3 ligases toward a specific substrate protein is influenced by many factors including the correct cellular location and enzyme stability (Weissman et al., 2011). Moreover, E3 activity is regulated via post-translational modifications both at the level of substrate or E3 enzyme (Metzger et al., 2012, 2014). Thus, mutations in many different domains within an E3 enzyme may ultimately influence levels of target proteins. Because instability domains in substrate proteins are generally recognized by multiple E3 ligases, mutations in the RING finger often lead to dominant-negative or gain-of-function effects, inhibiting the actions of the full lengths enzyme and/or other E3s, depending on the cellular context (Bach and Ostendorff, 2003). Indeed, forced expression of RING finger mutated RLIM in cells inhibits the functions of endogenous RLIM and likely other E3 ligases, leading to dramatically increased cellular levels of target proteins CLIM and HDAC2 (Ostendorff et al., 2002; Kramer et al., 2003), while levels of these proteins in *Rlim*KO cells only increase marginally (Shin et al., 2010).

Not surprisingly, mutations in genes encoding E3 ligases are often associated with human diseases mostly due to mis-regulation of substrate protein levels (Ciechanover and Schwartz, 2004), and mutations in *Rlim* have been associated with intellectual disability and autism (Tonne et al., 2015; Hu et al., 2016). However, while evidence suggests altered neuronal differentiation in mouse male ESCs lacking *Rlim/Rnf12* (Bustos et al., 2018), CNS development in mice lacking *Rlim* appears normal and thus, it is unclear at this stage whether human disability phenotypes are caused by the lack of functional RLIM protein, gain-of-function activity acquired by the mutated RLIM or a combination of both. Male mice systemically lacking *Rlim* are born at Mendelian ratios, appear healthy and grow up to be fertile (Shin et al., 2010, 2014; Wang et al., 2016) and even though minor functions cannot be excluded, these results indicate no general, major developmental function of *Rlim* in male mice. However, during female embryogenesis, major roles of *Rlim* have been identified in conjunction with Rex1 during X dosage compensation.

## Rlim/Rnf12, Rex1 and rXCI

In an attempt to validate a stochastic model of rXCI that postulates the existence of an X-linked activator of XCI (Monkhorst et al., 2008), *Rlim/Rnf12* was identified as an activator of XCI (Jonkers et al., 2009). Using the established F121 female ESC line, a KO ESC line was generated (Jonkers et al., 2009; Barakat et al., 2011), hereafter referred to as *Rnf12KO*. Indeed, upon differentiation, *Rnf12KO* ESCs failed to activate *Xist* transcription and initiate XCI, and in corresponding heterozygous ESCs rXCI was skewed toward inactivation of the KO allele (Jonkers et al., 2009; Barakat et al., 2011). Moreover, data from an *Rnf12KO* derived ESC line indicated that X-pairing was not needed for XCI (Barakat et al., 2014). Based on these results a model was proposed in which the dose of RLIM expressed from two alleles was required to trigger rXCI (Barakat et al., 2011). However, ESCs lacking *Rlim*, which were isolated from an independent mouse model containing a conditional *Rlim* KO allele (referred to as *RlimKO*) developed *Xist* clouds and initiated XCI upon differentiation *in vitro* and *in vivo*, and in tetraploid complementation experiments these ESCs were able to undergo rXCI and form post-gastrulation embryos (Shin et al., 2010, 2014). rXCI in the absence of *Rlim* in mice was supported by targeting the cKO of *Rlim* specifically in epiblasts via Sox2-Cre. Moreover, RLIM protein levels in WT embryos are downregulated specifically in differentiating epiblast cells at the timepoint of X-reactivation, shortly before the onset of rXCI (Shin et al., 2014). However, in contrast to differentiating epiblast cells, RLIM downregulation does not occur in ESCs differentiated *in vitro* (Wang et al., 2017). Thus, while *Rlim* is dispensable for rXCI *in vivo* and in female ESCs freshly isolated from embryos, it was required in the female F121 ESCs.

The identification of the pluripotency factor Rex1/Zfp42 as RLIM target (Gontan et al., 2012) has proven a major step in XCI regulation. Indeed, RLIM strongly interacts with and polyubiquitinates Rex1 in female ESCs leading to targeted proteasomal degradation. Rex1 acts as a potent repressor of *Xist* transcription both by repressing *Xist* directly in *trans* and in *cis* by activating the lnc*Tsix* (Gontan et al., 2012). Thus, the proteasomal targeting of Rex1 has provided a mechanism for *Rlim*’s positive action on XCI.

Two recent papers have provided much evidence not only that the dosage of nuclear Rex1 is critical in regulating the XCI process but have also shed light on the ESC controversy. One study generated various CRISPR/Cas9 mediated ESCs carrying an *Rlim* deletion in a Pgk12.1 (Norris et al., 1994) female ESC background (RlimKOp) (Wang et al., 2017). These cells were able to undergo XCI at much higher rates of efficiency when directly compared with *Rnf12KO* ESCs. It was discovered that the *Rnf12KO* did not represent a full knockout but that a 45kD truncated RLIM protein consisting of 333 amino acids (referred to as RLIM333) is expressed from the “KO” *Rlim* locus (Wang et al., 2017). As RLIM333 contains the NLS and a partial BD but lacks the NES and the RING finger (see Figure 1A), this protein can no longer shuttle but is trapped in the nucleus and consistent with the fact that Rex1 interacts with the BD, forced expression of RLIM333 in ESCs (*RlimKO* or WT) leads to nuclear accumulation of Rex1, indicating gain-of-function activity. Moreover, while *Rnf12KO* ESCs (*Rlim333f*) express Rex1 in the nucleus, cell derivatives with a CRISPR/Cas9-mediated deletion of RLIM333 (*Rlim0f*, in F121 background) are XCI competent at least to some degree and displayed mostly cytoplasmic Rex1 (Supplementary Figure 1). Together, these results indicate a direct connection between nuclear accumulation of Rex1 and the presence of RLIM333 and show that lack of *Rlim* in ESCs does not necessarily lead to nuclear accumulation of Rex1 (Wang et al., 2017). However, in a newly generated ESC line (in F121) that lacks the entire *Rlim* ORF (referred to as Rnf12^CR–/CR–^) and is unable to undergo XCI, XCI activity was restored upon additional deletion of Rex1 (Gontan et al., 2018). In contrast to ESCs examined in the first study, however, Rex1 accumulated in nuclei of Rnf12^CR–/CR–^ cells, presumably due to lack of E3 ligase activity (Gontan et al., 2018). These results are important as they demonstrate that in *Rnf12KO* it is Rex1 activity that prevents these cells from undergoing XCI. Thus, in various female ESC lines nuclear Rex1 accumulation is caused by the presence of RLIM333 or the lack of *Rlim*, while the latter does not affect Rex1 in all ESC lines.

The observed differences in Rex1 distribution in various ESCs models suggests the existence of additional pathways regulating both levels and subcellular localization of Rex1 that might include mechanisms affecting RLIM’s E3 ligase activity and/or differences in cellular competence factor repertoire. In this context it is important to mention that various ESC lines WT for *Rlim* display very different endogenous levels of Rex1, with Pgk12.1 ESCs expressing low but F121 and the male E14 (Hooper et al., 1987) lines much higher levels of Rex1, and at least endogenous Rex1 levels and cellular localization in Pgk12.1 are *Rlim*-independent (Wang et al., 2017). Moreover, ESC culture conditions may play an important role as physiological O_2_ levels improved XCI efficiency in *RlimKO* ESC lines (Wang et al., 2017). A powerful *Rlim*-independent cellular pathway regulating Rex1 is induced upon ESC differentiation, when Rex1 protein rapidly drops to undetectable levels even in the absence of *Rlim* (Gontan et al., 2012; Wang et al., 2017). Thus, one common feature among various ESC lines lacking *Rlim* is that nuclear exclusion of Rex1 strongly correlates with their ability to undergo XCI *in vitro*.

## Roles of Rlim/Rnf12 During iXCI

In contrast to rXCI, there is little controversy about the major role *Rlim* plays during iXCI. Indeed, female mice receiving a maternally transmitted *Rlim* KO allele (KOm) die (Shin et al., 2010) and not a single female receiving a KOm allele has been born in over 10 years of breeding. In contrast, females receiving a paternally transmitted KO allele (KOp) are born at Mendelian ratios and appear to undergo normal XCI. This parent-of-origin effect is a consequence of the fact that iXCI silences exclusively the Xp and the *Rlim* gene is X-linked. KOm females die around implantation due to defective development of trophoblast tissues caused by iXCI failure (Shin et al., 2010).

**FIGURE 2:**
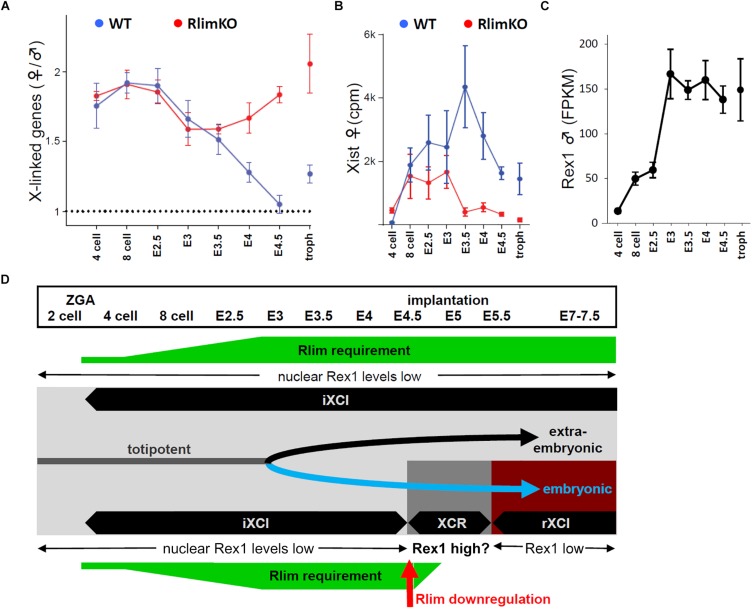
**(A–C)** Comparisons of gene expression profiles during preimplantation development as elucidated by single embryo RNA-seq. All data shown originate from the same dataset (RlimKO; Wang et al., 2017). Shown are comparisons of total X-linked gene expression in female RlimKO and WT embryos, normalized to males **(A)**, Xist RNA expression in RlimKO and WT females **(B)**, and Rex1 expression in males **(C)**. Note that once Rex1 reaches high levels at embryonic stage E3, Xist levels in RlimKO females drop dramatically at E3.5 halting/reversing the process of X dosage compensation. **(D)** Summary of XCI during female mouse embryogenesis. The requirement of Rlim (green) and its effect on nuclear Rex 1 protein levels are shown. Embryonic stages, iXCI, rXCI and XCR as well as cell lineages are indicated (gray, totipotent cell lineage; black, extraembryonic cell lineages; blue, embryonic/epiblast cell lineage).

Concerning the precise function during iXCI, a crucial role of *Rlim* in the maintenance of iXCI at blastocyst stages appears clear. This role was genetically established by inducing the *Rlim* cKO in embryos after ZGA via a paternally transmitted Rosa26-Cre transgene as well as by data obtained from RNA-seq experiments following expression profiles of X-linked genes and *Xist* transcription in single embryos across pre-implantation stages comparing *Rlim*KO with WT embryos (Figures 2A,B; Wang et al., 2016). Another mouse model that targets the *Rlim* gene has recently been generated based on *Rnf12KO* ESCs. Like *RlimKO* mice, females receiving a maternal *Rnf12KO* allele do not undergo iXCI leading to defective trophoblast development (Gontan et al., 2018). Strikingly, iXCI is rescued in female mice containing a *Rnf12/Rex1* double KO, establishing a genetic link between these two genes during iXCI. This result is exciting as it identifies Rex1 as the critical target of *Rlim* during iXCI *in vivo*. Moreover, Rex1 protein accumulates in nuclei of *Rnf12KO* blastocysts (Gontan et al., 2018). Indeed, in WT animals, Rex1 mRNA levels increase dramatically from 4 cell staged embryos to early blastocyst stages (Figure 2C; Wang et al., 2016), and Rex1 protein is detected in the cytoplasm of cells throughout mouse pre-implantation development, while it is undetectable in nuclei of cells in WT embryos up to morula stages (Climent et al., 2013). These data emphasize the critical role that *Rlim* plays in the nuclear exclusion of Rex1 during iXCI.

As for the role of *Rlim* in initiating *Xist* expression and iXCI, the results are less clear-cut. At early pre-implantation stages single embryo RNA-seq data indicate that the process of X dosage compensation initiates at least to some degree in females lacking *Rlim* but is then interrupted at early blastocyst stages (Figures 2A–C; Wang et al., 2016), when Rex1 levels increase. Formation of *Xist* clouds in a significant number of cells up to E2.5 was confirmed by RNA-FISH in *RlimKO* females. Importantly, the *RlimKO* embryos were generated using mothers systemically lacking *Rlim* including in their germline (Wang et al., 2016). Because the profiles of X-linked gene expression in *RlimKO* embryos mirror those observed in WT females at early pre-implantation stages (Figure 2A), this result argues against influences of maternally transmitted Rex1 which, like *Rlim* is expressed in oocytes (Climent et al., 2013), on initiation of iXCI in next generation embryos. However, when compared to WT, the number of cells lacking a *Xist* cloud is diminished in *RlimKO* females at all timepoints investigated (Shin et al., 2010; Wang et al., 2016). Therefore, while iXCI initiation is not absolutely dependent on *Rlim* at least in the C57Bl/6 genetic background, its presence likely promotes this process.

It is important to point out here that in the *Rnf12KO* system, embryos are expected to express the RLIM333 protein in all cells at pre-implantation stages, but this has not been examined. Because effects of RLIM333 expression and *Rlim* deletion on Rex1 are partially overlapping, additional repressive contributions of the truncated *Rlim* protein on the iXCI process cannot be excluded, and iXCI in *Rnf12KO* animals appears more severely inhibited when compared to *RlimKO* animals, especially during iXCI initiation (Gontan et al., 2018). Thus, while the *Rex1KO* in *Rnf12KO* mice removes the effects on XCI induced by both lack of *Rlim* and the presence of RLIM333, this model cannot distinguish what caused the nuclear accumulation of Rex1 protein.

## Discussion

The discovery of Rex1 as the crucial RLIM target during iXCI represents a major finding, assigning critical roles for the interplay of both genes in controlling the process of X dosage compensation in mice, in particular for iXCI and possibly XCR in epiblast cells. A general theme that emerges is that the major role of *Rlim* during XCI is to regulate nuclear Rex1 levels in order to enable *Xist* transcription. This is supported by genetic data in mice, Rex1 mRNA and protein expression during pre-implantation development and some data from ESC model systems. Incorporating these data in a previously formulated model (Wang et al., 2017) (see also Figure 2D), X dosage compensation in mice might be carried out in a scenario similar to that outlined below: Initiation of *Xist* transcription at the end of 2-/beginning of 4 cell-stage likely occurs under low levels of embryonic Rex1 (Figure 2C) and at least in part independent of *Rlim*. As Rex1 mRNA levels increase during pre-implantation development, the function/requirement of *Rlim* becomes more important to keep Rex1 protein excluded from nuclei of cells to ensure the continuation/maintenance of *Xist* transcription and the iXCI process. Thus, when *Rlim* is downregulated specifically in epiblasts of late blastocysts, it is likely that high Rex1 mRNA levels translate into rapid nuclear accumulation of Rex1 protein. Because Rex1 represses *Xist* but activates *Tsix* transcription, this spike in nuclear Rex1 likely facilitates the XCR process and/or contributes to an introduction/establishment of *Xist* regulation in *cis* via *Tsix* during rXCI *in vivo*. However, to ensure rXCI, Rex1 needs to be eliminated from nuclei of differentiating epiblast cells in a *Rlim*-independent manner, to allow for initiation of *Xist* transcription. The mechanisms for this are unclear but it is tempting to speculate the involvement of a pathway that is activated upon epiblast differentiation, partially mimicked in ESCs by LIF removal/embryoid body formation. *Xist* activation and rXCI in differentiating epiblast cells would then occur in the absence of both RLIM and Rex1. Conceptually, such a model shifts the critical dose dependency of the XCI process from *Rlim* to nuclear Rex1, as during iXCI *Rlim* is transcribed mostly from a single allele.

Concerning initiation of iXCI, it is important to point out that both RLIM and Rex1 proteins are detectable in the female germline (Shin et al., 2010; Climent et al., 2013, Wang et al., 2016). However, while initiation of iXCI in embryos occurs at least to some degree in the C57Bl/6 genetic background, this likely depends on maternal Rex1 levels, which might vary in individual females or in females of other genetic backgrounds. Thus, in early female embryos RLIM may serve as a safeguard of iXCI, protecting against maternal Rex1. Indeed, the ability of RLIM to target DNA-bound proteins (Ostendorff et al., 2002) is predicted to efficiently counteract repressive functions of both maternally transmitted and embryonic Rex1 on *Xist* regulatory sequences.

Concerning XCI in various female ESC models, the ability of female ESCs to undergo XCI correlates with low nuclear levels of Rex1. However, nuclear exclusion and overall cellular levels of Rex1 is controlled by RLIM in some ESC lines, but not in others, indicating the existence of alternative pathway(s) that may be sensitive to culture and/or differentiation conditions and possibly also involving multiple mechanisms on RLIM regulation such as E3 ligase activity and differences in the epigenetic make-up selected via clonal line selection. Moreover, various ESC lines might represent different developmental states with possibly leaky suppression of *Rlim*-independent pathway(s) controlling Rex1, thereby explaining different baseline levels and localization of Rex1 protein in cells. Thus, while RLIM appears the only factor controlling nuclear exclusion of Rex1 at early pre-implantation stages, in ESCs *Rlim* represents only one of multiple pathways. These data suggest that the dynamics of both *Rlim* and Rex1 regulation during mouse development is not always paralleled in ESCs in culture, and more work is needed to elucidate the cellular regulation of Rex1 protein.

Concerning RLIM333, the presence of this truncated protein in *Rnf12KO*-derived systems is based on a cloning artifact. RLIM333 causes nuclear accumulation of Rex1, an effect overlapping with that of the *RlimKO* and thus, this gain-of-function activity will be eliminated by an additional Rex1KO. However, because RLIM333 lacks specific protein domains, its activity is no longer associated with pathways regulating the full-length protein in terms of expression, stability and E3 ligase activity. In *Rnf12KO*-derived model systems including mice, there is likely enhanced effects on nuclear Rex1 due to the lack of RLIM and the presence of RLIM333, potentially influencing various aspects of the iXCI process in a temporary, qualitative and quantitative manner, and the effects of RLIM333 on XCI cannot be distinguished from those induced by the lack of *Rlim*. Outside the *Rex1-Rlim* axis, RLIM has other E3 ligase targets most of which are also targeted by other E3s. While the KO has little/no effect on such targets, the presence of RLIM333 might, and thus other phenotypes are likely. This is why the reported lethality in male *Rnf12KO* (Gontan et al., 2018) is concerning as in *RlimKO* models including germline *RlimKO* models (Cre-recombinase negative) no lethality has been observed in males lacking RLIM over more than 10 years. Therefore, data obtained by the *Rnf12KO* model need to be interpreted not only from the KO perspective, but also from a gain-of-function perspective. Because the terms “KO” or “−/−” are incorrect and cause confusion, derivatives of the *Rnf12KO* ESCs should be renamed to account for the presence of RLIM333.

In summary, recent results have assigned a crucial importance to the *Rlim-Rex1* axis in controlling XCI both *in vivo* and *in vitro*. Going forward, it will be important to elucidate the functional evolutionary conservation of this module in other species, the dynamics between both factors specifically in epiblast cells during implantation including effects on *Xist/Tsix*, and to identify factors that mediate rXCI *in vivo*.

## DATA AVAILABILITY STATEMENT

All datasets generated for this study are included in the article/Supplementary Material.

## ETHICS STATEMENT

The animal study was reviewed and approved by UMMS Institute of Animal Care and Usage Committee.

## AUTHOR CONTRIBUTIONS

Both authors listed have made a substantial, direct and intellectual contribution to the work, and approved it for publication.

## Conflict of Interest

The authors declare that the research was conducted in the absence of any commercial or financial relationships that could be construed as a potential conflict of interest.
