# Supplementary figures and images for: Rlim/Rnf12, Rex1, and X Chromosome Inactivation
Source: Front Cell Dev Biol. 2019 Oct 31;7:258. doi: 10.3389/fcell.2019.00258 (PMC6834644; doi:10.3389/fcell.2019.00258)

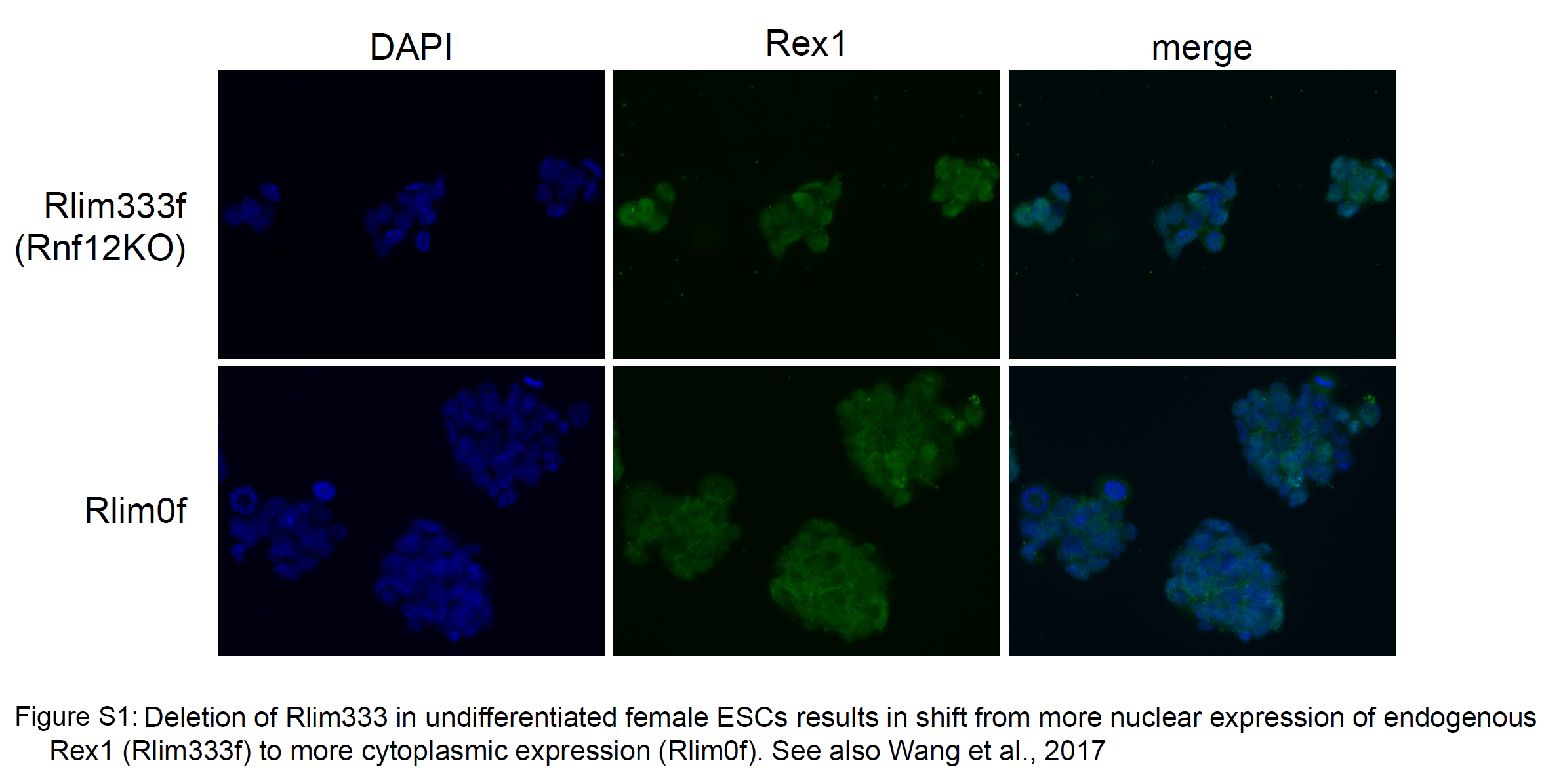

Supplement: Supplementary file 1 [file Image_1.tif]
